# Quality of diabetes care worldwide and feasibility of implementation of the Alphabet Strategy: GAIA project (Global Alphabet Strategy Implementation Audit)

**DOI:** 10.1186/1472-6963-14-467

**Published:** 2014-10-11

**Authors:** James D Lee, Ponnusamy Saravanan, Lakshminarayanan Varadhan, John R Morrissey, Vinod Patel

**Affiliations:** Diabetes and Endocrinology Centre, George Eliot Hospital NHS Trust, College Street, Nuneaton, Warwickshire, CV10 7DJ UK; Warwick Medical School, University of Warwick, Coventry, UK; University Hospital North Staffordshire, Stoke on Trent, UK

**Keywords:** Diabetes mellitus, Chronic disease management, Alphabet strategy, Quality of care, Checklist, Multifactorial intervention

## Abstract

**Background:**

The Alphabet Strategy (AS) is a diabetes care checklist ensuring “important, simple things are done right all the time.” Current audits of diabetes care in developed countries reveal wide variations in quality with performance of care processes frequently sub-optimal. This study had three components:

- an audit to assess diabetes care quality worldwide,
- a questionnaire study seeking opinions on the merits of the AS,
- a pilot study to assess the practicality of implementation of the AS in a low socioeconomic setting.

**Methods:**

Audit data was collected from 52 centres across 32 countries. Data from 4537 patients were converted to Quality and Outcome Framework (QOF) scores to enable inter-centre comparison. These were compared to each country’s Gross Domestic Product (GDP), and Total Health Expenditure percentage per capita (THE%). The opinions of diabetes patients and healthcare professionals from the diabetes care team at each of these centres were sought through a structured questionnaire. A retrospective audit on 100 randomly selected case notes was conducted prior to AS implementation in a diabetes outpatient clinic in India, followed by a prospective audit after four months to assess its impact on care quality.

**Results:**

QOF scores showed wide variation across the centres (mean 49.0, range 10.2–90.1). Although there was a positive relationship between GDP and THE% to QOF scores, there were exceptions. 91% of healthcare professionals felt the AS approach was practical. Patients found the checklist to be a useful education tool. Significant improvements in several aspects of care as well as 36% improvement in QOF score were seen following implementation.

**Conclusions:**

International centres observed large variations in care quality, with standards frequently sub-optimal. 71% of health care professionals would consider adopting the AS in their daily practice. Implementation in a low resource country resulted in significant improvements in some aspects of diabetes care. The AS checklist for diabetes care is a freely available in the public domain encompassing patient education, care plans, and educational resources for healthcare professionals including summary guidelines. The AS may provide a unique approach in delivering high quality diabetes care in countries with limited resources.

**Electronic supplementary material:**

The online version of this article (doi:10.1186/1472-6963-14-467) contains supplementary material, which is available to authorized users.

## Background

Diabetes is a growing problem predicted to reach epidemic proportions. The WHO forecasts the number of people living with diabetes will reach 592 million by 2035, with no country being exempt from the rising tide. It is expected that low and middle income countries will bear most of this increase [1]. The estimated worldwide healthcare expenditure for managing diabetes and its complications totalled at least $US 548 billion in 2013, equating to an average of $US 1437 per person with diabetes. However, there is considerable variation in spending between regions and countries. For example, Norway spent an average of $US 10,368 per person with diabetes, compared to under $US 30 in those living in Somalia. It is not clear how much variation exists in the quality of diabetes care and its relationship to resources.

There is currently a large evidence base to guide the optimal management of diabetes to prevent complications [2]. Disappointingly, there remains frequent sub-optimal performance of diabetes care processes and control of cardiovascular risk factors [3, 4]. The National Diabetes Audit of England and Wales show that in those with Type 1 and Type 2 diabetes, 43% and 63% respectively received eight out of the nine National Institute for Health and Care Excellence (NICE) recommended annual care processes (urinary albumin, eye screening, foot exam, smoking review, body mass index, cholesterol, blood creatinine, HbA1c, and blood pressure). These completion rates were slightly lower than those from the previous year [5]. The provision of high quality diabetes care requires patient-centred, evidence-based management of multiple risk factors delivered by professional multidisciplinary teams. However specialist multidisciplinary teams are expensive and not cost effective especially in low and middle income countries [6].

A checklist can be considered a list of key reminders of critical or important steps in performing a process with the aim of ensuring safe and effective completion of the task. They have been employed successfully in aviation and manufacturing control as a simple tool for reducing human error and ensuring best practice adherence. Their transition into the healthcare arena has been limited, but in areas where they have been employed in medicine, their implementation has been associated with significant improvements in patient safety [7, 8]. Following the publication of these papers, there has been growing interest in checklists or care bundles as a possible solution to improving care quality and reducing clinical risk. However, attributing all the ground-breaking changes to the simple use of a checklist infers the wrong conclusion. Despite summarising and simplifying the technical aspects of a procedure, this technical solution even if based on the latest rigorous evidence has to counter innate resistance from healthcare professionals that is often social and cultural [9]. Merely providing the checklist to institutions did not result in instant results. Rather, months of painstaking work to organisational systems and personnel were some of the necessary aids in successful implementation of the checklists and their use.

The Alphabet Strategy is diabetes care based on a mnemonic [10]:

A- Advice, regarding smoking avoidance, ideal weight attainment, sensible dietary choices, and regular physical activity, with specific individualised advice such as ‘flu vaccination
B- Blood pressure, with individualised targets according to guidelines and co-morbidities
C- Cholesterol assessment, with targets individualised according to co-morbidities
C- Creatinine/microalbuminuria assessment
D- Diabetes glycaemic control with an individualised HbA1c and avoidance of hypoglycaemia
E- Eye examination, conducted at least yearly, with appropriate timely referral if indicated
F- Foot examination, conducted at least yearly, with appropriate timely referral if indicated
G- Guardian drugs: appropriate use of drugs protective against CVD and other complications particularly aspirin, ACE inhibitors or ARBs, and statins.

It can be considered a checklist or aide-mémoire designed to address all the essential components of effective diabetes care, ensuring “important, simple things are done right all the time [11].” Its use has promoted collaborative patient management across primary and secondary care, allowed multidisciplinary team working, and encouraged patient involvement and empowerment, using a patient education programme based around the checklist. This framework has been used as an audit template, in written correspondence, and in education of healthcare professionals and patients.

It has been successfully implemented in the diabetes clinics at a District General Hospital in the UK (George Eliot Hospital, Nuneaton) where it has resulted in high quality diabetes care evidenced by its clinical effectiveness evaluation [12] and as reflected in the UK Quality and Outcomes Framework (QOF) scores from general practice surgeries using the checklist [13]. It has also been shown to be an effective tool in achieving randomised clinical trial standards in routine clinical practice [14].

The Alphabet Strategy is a freely available, non-copyright concept, accessible in the public domain to allow adaption to local circumstances. It could provide high quality diabetes care at minimal cost. This might be particularly useful in low to middle income countries where “non-specialists” health care professionals deliver the majority of diabetes care. It is not known whether such a checklist is suitable and adaptable by non-specialist health care professionals outside the UK.

The Global Alphabet Strategy Implementation Audit Project (GAIA) consisted of three components:

- Audit of diabetes care using the Alphabet Strategy as a template to determine the quality of diabetes care at various centres worldwide,
- A qualitative questionnaire study to determine the feasibility of implementation of the Alphabet Strategy in various healthcare settings,
- Pilot study of implementation of the Alphabet Strategy in India in a low resource setting.

## Methods

### Worldwide audit of diabetes care

#### Collection of data

A retrospective audit was conducted within a nine-month period in 52 secondary and tertiary hospital centres across 32 countries. Data was collected on processes of diabetes care according to the Alphabet Strategy checklist template (numerical values and documentation of care process within the previous 15 months), demographics and complications. This template is illustrated in Alphabet Strategy checklist template used in GAIA.

**Alphabet Strategy checklist template used in GAIA**

- Advice - weight, BMI, smoking status
- Blood pressure
- Cholesterol - lipid profile where available
- Creatinine and microalbuminuria, or proteinuria where microalbuminuria is not available
- Diabetes control by HbA1c, or fasting or post-prandial glucose where HbA1c not available
- Eye examination - evidence of fundus exam either by direct ophthalmoscopy or retinal photographs
- Foot examination
- Guardian drug usage - aspirin, ACE inhibitor or angiotensin receptor blocker, statins

Data collection was performed by final year medical students from Warwick Medical School, Coventry, England during their electives. The elective is a well established period in the undergraduate curriculum where students can travel abroad to gain experience in overseas healthcare systems. Students arranged their own destination locations and required approval as a suitable centre from Warwick Medical School.

Letters of introduction about the concept signed by VP and JRM were sent to participating diabetes centres seeking permission to conduct the audit and questionnaire studies. All students underwent basic training in auditing notes. An Alphabet Strategy pack containing all relevant materials was provided before they embarked to their destination centres (CD containing lecture presentation on the Alphabet Strategy checklist, consultation videos, all relevant published papers regarding the Alphabet Strategy, clinic consultation template and data collection forms).

The notes to be audited were randomly selected by the host diabetes care team at each hospital. The host care teams were blinded to the assessment parameters of the audit. On their return from their elective locations, each medical student was required to sign a declaration of the authenticity of the data collected.

#### Statistical analysis

Data pertaining to care process performance and target process achievement from each centre was converted into QOF scores to quantify care quality and enable comparison of results with other hospitals. QOF is a voluntary annual award and incentive scheme open to all general practice surgeries in England aiming to provide resources and rewarding good quality of care. Practices score points according to their achievement against certain quality indicators in a variety of disease conditions. The total score attained is correlated with the degree of financial reward with a higher score achieving greater monetary remuneration. The parameters of the original version of QOF were employed for score calculation [15]. The use of QOF scoring allowed the combined assessment of diabetes care process performance and intermediate outcome measures such as degree of blood pressure control and HbA1c. The QOF quality indicators for diabetes and their associated maximum achievable scores are shown in Table 1.

**Table 1:** **QOF targets and maximum points achievable (2004), with format adapted to domains of the Alphabet Strategy**

Quality Indicator			Maximum points†
General	1	General background data: register of all diabetes mellitus patients	6
Advice	2	% whose notes record diabetes mellitus	3
	3	% with record of smoking status	3
	4	% who smoke and a record that smoking cessation advice has been offered	3
	18	% who have had influenza immunisation in the preceding 1 Sep to 31 Mar	5
Blood pressure	11	% with a record of blood pressure	3
	12	% whose last blood pressure is 145/85 or less	17
Cholesterol & creatinine	16	% who have a record of total cholesterol	3
	17	% whose last total cholesterol is 5 or less	6
	13	% with a record of microalbuminuria testing	3
	14	% with a record of serum creatinine testing	3
Diabetes control	5	% who have a record of HbA1c	3
	6	% with HbA1c of 7.4 or less	16
	7	% with HbA1c of 10 or less	11
Eyes	8	% who have a record of retinal screening	5
Feet	9	% with a record of presence or absence of peripheral pulses	3
	10	% with a record of neuropathy testing	3
Guardian drugs	15	% with proteinuria or microalbuminuria who are treated with ACE inhibitor (or ARB)	3
Total points			99

The numbers in this column denotes the quality indicator in the original QOF Quality Indicator list.

†Points are given from a specific lower threshold towards a higher threshold.

Provisions in the QOF framework to permit exemption of certain patients from analysis were not applied. The achievement of NICE nine key recommended processes of diabetes care was also assessed.^5^

The performance of the various centres according to their QOF scores were compared to their country’s corresponding Gross Domestic Product (GDP), Total Health Expenditure percentage per capita (THE%), and life expectancy from birth (LE). The World Bank Gross National Income (GNI) per capita operational guidelines and analytical classifications was employed to classify countries according to low, lower middle, upper middle and high incomes. GDP, GNI per capita, THE% and LE data were obtained from the World Bank website (data.worldbank.org) for the years corresponding to the audit. Statistical analysis was performed using Wizard for Mac (version 1.4.3).

### Questionnaire study

#### Collection of data

A qualitative questionnaire study was performed at each of the destination election locations to determine opinions regarding usability and utility of the Alphabet Strategy from healthcare professional and patients.

A tutorial on the Alphabet Strategy checklist was presented by the medical students to the host diabetes care team. The presentation included data on the evidence base for each of its components, evidence for its effectiveness in the UK, and patient education posters. Self-administered questionnaires were then completed by each member of the team present. A random selection of patients were taken through the Alphabet Strategy and the education posters. Their opinions on the checklist were also sought with the same questionnaire through in-person interview. Language translators were employed in the presentation and for questionnaire completion as necessary. Students were advised to aim for at least five completed questionnaires.

#### Data analysis

The structured questionnaire is shown in Additional file 1. The first nine questions were closed and answered on a visual analogue scale (VAS). Questions 10 and 11 were dichotomous response formats. Question 12 was a closed format question allowing multiple answers from a choice of six responses. Question 13 consisted of two open questions. The questionnaire was previously designed, planned and piloted by the Diabetes Care Team in collaboration with patients at George Eliot Hospital NHS Trust, Nuneaton, Warwickshire, UK.

Results on the 10 cm VAS were converted into an equivalent numerical value between 0 to 100, to the nearest whole figure. Values between 0–50 were considered to answer the question in the negative (disagree or not useful), whereas those greater than 50 to 100 implied agreement. Mean, median, mode, interquartile range, and range were calculated. Unanswered responses were excluded from statistical analysis.

### Implementation of the alphabet strategy in a non-high income country

The Alphabet Strategy has been implemented successfully in our Diabetes Centre. Compared to many developing countries, England is relatively resource plenty. It was uncertain whether the Alphabet Strategy could be implemented in countries with limited healthcare spending per capita. A pilot study was therefore undertaken to determine whether the checklist could be implemented in Shenbagam, Madurai, India. In this town, a colleague of LV ran a private outpatient clinic providing diabetes care to the local population. Dr V Palanikumaran was the sole diabetologist. His other staff consisted of a receptionist, a dietician, and an auxiliary nurse. The clinic would see at least 30 diabetes patients per day, with a 50:50 ratio of new/follow-up people. The local population numbered approximately 200,000 people primarily of low socioeconomic status. This diabetes clinic was one of several that were available to the community.

Dr V Palanikumaran and his staff were fully briefed on the concept of the Alphabet Strategy and agreeable to its implementation in his clinic. A pre-implementation audit was performed on 100 randomly selected case notes to determine baseline completeness of diabetes evaluation. Data was collected based on the Alphabet Strategy checklist with modifications to adapt to the limited local resources: estimation of HbA1c was replaced by measurement of fasting and postprandial blood glucose, microalbuminuria testing was replaced by testing for proteinuria with dipstix, and diabetic retinopathy screening as replaced with fundus examination. HbA1c, microalbuminuria, and retinal photography were not done routinely as they were expensive.

Following implementation of the Alphabet Strategy, a prospective audit of 100 consecutive patients was undertaken four months later using the checklist template to determine diabetes care quality. QOF scores were calculated for both groups. Changes in the proportion of people receiving care processes were assessed statistically using chi-squared test.

All components of the study was considered by the Research and Development department at George Eliot Hospital to be an audit of care quality and implementation of good practice guidelines such as those from the EASD, ADA, and IDF. Ethical approval was therefore not deemed necessary following this advice and consultation.

## Results

### Worldwide audit of diabetes care

Students returned from their elective placements with audit data from all their centres. Those centres contributing less than 50 patients for the audit were excluded from analysis. Data was therefore available on 4537 people with diabetes, from 45 centres across 28 countries, covering all continents except Antarctica. Mean age was 57 years, with a median and interquartile range (25-75th centile) of 58 and 48–68 years respectively. Of the 44 centres with age data, the distribution of ages in 18 centres followed a normal distribution by the Shapiro-Wilk test. The remaining centres showed a negative skew. Comparison of median ages from each centre’s study cohort suggested unequal medians (Kruskal-Wallis test, P < 0.001).

People with type 2 diabetes comprised 83% of the total cohort. 12% were type 1 and missing data on diabetes type was 5%. 53% of the population studied were females.

QOF scores for each centre with their country’s respective GDP, THE% and LE are shown in Table 2. There was wide distribution in QOF scores across centres reflecting variability in care process performance and target process achievement. QOF scores followed a bimodal distribution pattern with the major and minor modes being 36 and 66 respectively. Mean and median were 48.97 and 41.29 respectively, with an interquartile range of 31.66-72.08. Average QOF scores were higher in European countries than in those representing the rest of the world ((mean QOF ± SD)76.3 ± 12.7 vs. 41.2 ± 19.7; p < 0.001).

**Table 2:** **QOF scores of centres with corresponding GNI, GDP, THE%, and LE**

Serial number	Country	QOF score (out of 99)	GNI per capita 2005/06*†	GDP per capita/US$*	THE%*	LE/years*
GRE2	Greece	90.14	H	23506	9.7	79
JER	Jersey	87.52	H	57000	8.1	80
FRA1	France	85.02	H	33819	11.1	80
SPA2	Spain	81.65	H	28025	8.3	81
IND10	India	80.56	LM	820	4.0	64
ENG	England	78.95	H	40342	8.5	79
FAL	Falklands	78.92	H	40342	8.5	79
FRA2	France	77.19	H	35457	11.1	81
AUS3	Australia	76.93	H	34149	8.4	81
NOR	Norway	76.54	H	65767	9.1	80
SPA	Spain	72.44	H	26056	8.3	80
AUS4	Australia	72.08	H	34149	8.4	81
AUS2	Australia	68.30	H	34149	8.4	81
GRE1	Greece	68.12	H	21621	9.6	79
IND1	India	66.79	LM	732	4.0	63
USA	USA	66.40	H	42516	14.7	77
SAF1	South Africa	59.05	UM	5235	8.8	51
BAR	Barbados	54.46	H	11109	7.0	76
IND8	India	53.48	LM	820	4.0	64
IND7	India	50.74	LM	820	4.0	64
MAL	Malaysia	48.31	H	5286	4.1	73
IRE	Ireland	45.69	H	48866	7.5	78
JAM	Jamaica	41.29	UM	4263	4.1	71
GUY	Guyana	37.74	LM	1949	6.0	68
WSA	Western Samoa	37.21	UM	2287	5.5	71
IND9	India	36.59	LM	5468	8.5	64
SAF2	South Africa	36.59	UM	820	4.0	51
IND5	India	36.16	LM	732	4.0	63
BAN	Bangladesh	35.71	LM	429	3.2	67
ZIM	Zimbabwe	35.12	L	434	8.1	45
IND3	India	33.91	LM	732	4.0	63
STK	St. Kitts	32.75	H	10394	5.4	71
GHA	Ghana	32.42	LM	495	7.1	61
PER	Peru	31.66	UM	3312	4.5	73
MAU	Mauritius	30.05	UM	5054	4.6	72
GRN	Grenada	29.26	UM	6804	6.4	75
IND4	India	29.20	LM	732	4.0	63
IND2	India	27.11	LM	732	4.0	63
SOL	Solomon Islands	25.26	LM	881	4.6	65
GHA2	Ghana	19.70	LM	495	7.1	61
AUS1	Australia	19.66	H	34149	8.4	81
IND6	India	17.27	LM	732	4.0	63
EGY	Egypt	16.16	UM	1209	5.2	72
BEL	Belize	13.35	UM	3821	3.5	75
TON	Tonga	10.16	UM	2573	4.7	71

*Gross National Income (GNI) per capita, Gross Domestic Product (GDP), Total Health Expenditure percentage per capita (THE%) and life expectancy from birth (LE) taken from data.worldbank.org for years 2005/2006.

†H: High income country; UM: upper middle; LM: lower middle; L: low.

Using Fisher transformation test, there was a positive correlation between QOF scores and GDP per capita (z = 5.233, r^2^ = 0.447, p < 0.001), QOF and THE% (z = 4.452, r^2^ = 0.355, p < 0.001), and QOF with LE (z = 3.371, r^2^ = 0.288, p < 0.001).

Table 3 shows the percentage of people receiving the NICE nine key processes of diabetes care (NICE 9). This set of key processes comprises five risk factor assessments (weight (BMI), smoking, blood pressure, serum cholesterol and glucose levels (HbA1c)) and fours tests for early complications (eye exam, microalbuminuria (or urine protein), serum creatinine and foot exam). Results have been font coded according to the process achievement of the cohort: bold ≥ 90%, italic 70-89%, normal font <70%. The achievement of the NICE 9 was positively correlated with THE% (Fisher transformation test, z = 3.284, p < 0.001). The correlation of the individual NICE 9 components to THE% is shown in Table 4. THE% appears to show positive correlation to the assessment of BMI, total cholesterol, HbA1c, creatinine, microalbuminuria, and eye and foot exams.

**Table 3:** **Recording of NICE nine key processes of care/% of centre cohort**

Serial no.	Country	QOF score	NICE 9	BMI*	Smoking*	BP*	TC*	Cr*	Microalb*	Urine prot*	HbA1c*	Eye* exam	Foot* exam
GRE2	Greece	90.14	60	**95**	*75*	**100**	**98**	**98**	**95**	**95**	**100**	**96**	**96**
JER	Jersey	87.52	74	**96**	**94**	**91**	**92**	**90**	*86*	54	**98**	**90**	**93**
FRA1	France	85.02	47	*87*	*78*	**97**	**94**	**93**	*83*	46	**95**	**94**	**92**
SPA2	Spain	81.65	6	**92**	8	**96**	**98**	*88*	61	6	**95**	*88*	*86*
IND10	India	80.56	0	**99**	**99**	**99**	**99**	49	0	**93**	0	**100**	**100**
ENG	England	78.95	3	60	**93**	**100**	*77*	**94**	10	4	**95**	69	*85*
FAL	Falklands	78.92	0	**98**	**94**	**98**	**98**	10	*84*	*88*	**90**	6	36
FRA2	France	77.19	63	**98**	**94**	**97**	**96**	**98**	59	*73*	**98**	**90**	**94**
AUS3	Australia	76.93	0	0	**90**	**96**	**98**	*84*	68	18	**100**	**100**	**100**
NOR	Norway	76.54	26	56	59	**100**	**98**	**100**	*84*	61	**99**	*84*	*83*
SPA1	Spain	72.44	16	*82*	66	*83*	*84*	*84*	62	28	*78*	55	41
AUS4	Australia	72.08	0	20	40	**95**	*86*	*72*	1	0	**94**	**100**	**98**
AUS2	Australia	68.30	4	31	35	*84*	*84*	66	20	2	**94**	55	54
GRE1	Greece	68.12	26	**92**	45	**100**	**100**	**100**	**99**	**99**	**100**	*77*	**90**
IND1	India	66.79	37	**98**	38	**93**	**100**	**98**	**97**	**98**	**99**	**95**	**95**
USA	USA	66.40	5	*77*	37	**95**	37	*78*	67	13	**97**	*88*	68
SAF1	South Africa	59.05	39	61	68	**99**	*88*	**97**	*86*	37	**97**	*84*	*88*
BAR	Barbados	54.46	0	68	**90**	**95**	*82*	7	0	17	*87*	**93**	*72*
IND8	India	53.48	0	45	6	**99**	*76*	**98**	46	*81*	57	*71*	*87*
IND7	India	50.74	1	**98**	46	**99**	22	*89*	0	*74*	2	**90**	**91**
MAL	Malaysia	48.31	2	26	51	**100**	*87*	*87*	19	59	61	65	66
IRE	Ireland	45.69	5	33	38	*71*	**93**	**94**	*82*	0	**93**	**96**	27
JAM	Jamaica	41.29	1	56	6	**97**	62	55	60	57	56	56	51
GUY	Guyana	37.74	0	0	5	**97**	31	42	1	5	0	0	0
WSA	Western Samoa	37.21	0	0	39	**97**	39	49	0	35	26	56	52
IND9	South Africa	36.59	0	10	*78*	**99**	40	*88*	10	9	51	40	63
SAF2	India	36.59	0	43	**90**	**100**	38	57	32	0	3	10	51
IND5	India	36.16	0	1	50	**95**	*80*	*79*	7	47	23	23	4
BAN	Bangladesh	35.71	0	**98**	**95**	**99**	10	14	65	3	8	0	0
ZIM	Zimbabwe	35.16	0	**100**	2	**100**	0	0	0	42	1	48	22
IND3	India	33.91	0	4	50	**100**	48	*86*	0	54	8	4	12
STK	St. Kitts	32.75	0	2	0	*81*	*76*	49	2	49	0	1	2
GHA	Ghana	32.42	0	**90**	**100**	**100**	5	21	2	6	1	22	8
PER	Peru	31.66	0	64	0	**90**	50	34	1	9	3	1	1
MAU	Mauritius	30.05	0	2	28	**96**	5	**93**	5	1	1	18	14
GRN	Grenada	29.26	0	1	*84*	**100**	19	*84*	10	6	0	13	3
IND4	India	29.20	0	*73*	**96**	**99**	*81*	27	1	0	6	**90**	69
IND2	India	27.11	0	0	0	**91**	0	49	4	4	0	4	2
SOL	Solomon Is	25.26	0	8	0	*74*	0	0	0	0	0	0	2
GHA2	Ghana	19.70	0	0	0	**100**	59	43	1	*80*	37	54	60
AUS1	Australia	19.66	0	60	*73*	17	48	39	18	0	47	29	41
IND6	India	17.27	0	0	3	**97**	13	19	20	15	0	1	0
EGY	Egypt	16.16	0	0	25	*86*	3	30	0	3	0	3	17
BEL	Belize	13.35	0	0	2	**98**	42	40	0	20	0	2	10
TON	Tonga	10.16	0	26	44	**96**	6	20	0	0	0	0	46

*Bold font >90; Italic font 70–89; Normal font <70.

**Table 4:** **Results of correlation of individual NICE9 components versus THE% using fisher transformation**

NICE9 care process assessment	Correlated?	Correlation coefficient	z statistic	p value
**BMI**	Yes	0.357	2.421	0.015
**Smoking**	No	0.282	1.877	0.061
**BP**	No	-0.081	0.528	0.598
**Cholesterol**	Yes	0.376	2.564	0.01
**Creatinine**	Yes	0.343	2.314	0.021
**Microalbuminuria**	Yes	0.493	3.496	<0.001
**Urine protein dipstix**	No	0.069	0.448	0.654
**HbA1c**	Yes	0.713	5.789	<0.001
**Eye exam**	Yes	0.491	3.484	<0.001
**Foot exam**	Yes	0.453	3.166	0.002

### Questionnaire study

Of the 52 host centres, completed questionnaires were returned from 44 units across 27 countries, giving a completion ratio 86%. Data from 203 questionnaires were available for analysis. 21% of the forms were completed by doctors. Nurses and patients comprised 10% and 16% respectively. The remaining 53% could not be categorised.

The average number of questionnaires per centre was 4.6, with a median and mode of 5. The interquartile range was 3–5. Eight centres did not complete a questionnaire. These were in the countries of Barbados, India, Jersey, South Africa, Sri Lanka, and St. Kitts.

#### Questions 1–9

**Figure 1:**
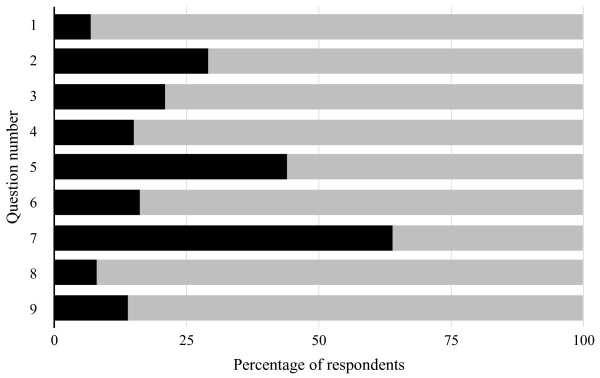
**Graphical representation of response to questions 1–9 on VAS.** Black ≤50; Grey >50.

Table 5 shows the results of question 1–9 using the VAS. As can be seen in Figure 1, the majority of respondents replied in the positive to questions 1–7 and 9. 64% answered question 7 in the negative.

**Table 5:** **Results of questions 1–9 using VAS format**

	Question	Total complete answers	Mean	Median	Mode	Interquartile range	Min range	Max range	% response >50
**1**	**What do you think of the alphabet strategy approach?**	203	83	87	100	77-96	2	100	93
**2**	**Are you likely to adopt this strategy in your clinical practice?**	192	69	80	100	48-92	0	100	71
**3**	**Do you think your patients would understand and benefit from the educational potential of this strategy?**	199	73	82	100	56-93	0	100	79
**4**	**Do you think this will improve the outcome of your practice?**	191	77	82	100	64-95	0	100	85
**5**	**Do you think this strategy can be applied to your economic background?**	199	57	60	100	33-88	0	100	56
**6**	**Do you think this strategy needs to be translated into your local language?**	195	78	91	100	78-98	0	100	84
**7**	**Do you think the English version would be more useful in your clinics?**	195	42	40	0	5-79	0	100	36
**8**	**What do you think of the diabetes care plan?**	201	83	87	100	76-97	5	100	92

#### Question 10–12

Responses to questions 10 and 11 are shown in Table 6. The Alphabet Strategy was thought to be practical and evidence-based by 91% and 98% of participants respectively.

**Figure 2:**
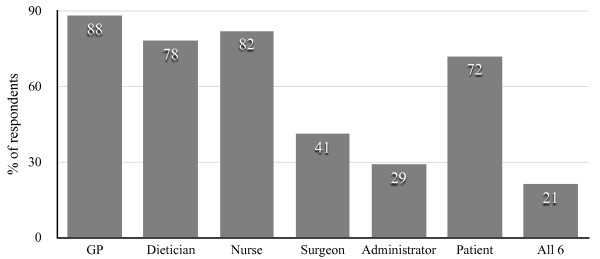
**Responses for question 12 (“Which healthcare professional should adopt the strategy?”).**

The bar chart featured in Figure 2 illustrates the responses for question 12. There were an average of 3.9 responses out of the six available that were selected by participants. The two most common responses were GP and nurse. 72% of replies answered that patients should adopt the strategy. Indeed of the 32 known patient respondents, 22 (69%) thought that patients should use the strategy.

**Table 6:** **Responses to questions 10 and 11**

	Question	Yes	No	Total respondents	% respondents answering yes
**10**	Do you think this strategy is practical?	179	18	197	91%
**11**	Do you think this strategy is evidence based?	176	4	180	98%

#### Summary of responses to question 13

##### General comments

Respondents thought the Alphabet Strategy was a good, simple, comprehensive guide and an aide memoir in a busy diabetes clinic. The posters were considered a useful tool for education of patients, though some replied that rural inhabitants would not understand the information as much as city dwellers. It would obviously require translation to the local language. There were comments on the difficulty and expense of HbA1c and cholesterol assessment, as well as the cost of medicine which would be paid for by the patient. Interestingly, a comment stated that exercising was not part of the culture of Tanzania.

##### How could this strategy be implemented?

Quite a few comments reiterated the need to translate the posters/strategy to a local language. The next most frequent comments were on the subject of education. Education and motivation of patients was a common theme, as well as education of healthcare professionals. There were comments about involving healthcare professionals in promoting and using the strategy, as well as being involved in patient education. Ease of access to the information for patients and doctors was mentioned by several respondents including leaflets/booklets, displaying posters in public buildings, doctors’ surgeries, pharmacies, the internet, and even modern media such a radio or TV. Several people highlighted again the high local economic costs of medicine to the patient and the cost of laboratory tests. There would need to be modifications to the strategy to meet local needs, for example simplification of the education posters for rural communities with low literacy rates, and use of fasting blood sugar instead of HbA1c.

A sample of the comments are included in Additional file 2.

### Implementation of the alphabet strategy in a non-high income country

The results of this implementation process are shown in Table 7, with the percentage of people documented to have received diabetes care performance checks before and after introduction of the Alphabet Strategy checklist tabulated. There was significant improvement in the documentation of total cholesterol, serum lipid profile, renal function and proteinuria, glycaemia and uptake of guardian drugs (p < 0.001). QOF scores increased by 36% from 45 to 61 (p < 0.001).

**Table 7:** **Changes to performance and documentation of diabetes care process following implementation of the alphabet strategy in India**

	Care process recording	% Before implementation	% After implementation	p value
**A**	Body Mass Index	99	99	NS
	Smoking status	99	99	NS
	Smoking cessation	100	100	NS
**B**	Blood pressure	99	99	NS
**C**	Total cholesterol	60	99	<0.001
	Lipid profile	10	64	<0.001
	Creatinine	5	49	<0.001
	Proteinuria	48	93	<0.001
**D**	Fasting and postprandial glucose	41	97	<0.001
**E**	Eye examination	98	100	NS
**F**	Feet examination	95	100	NS
**G**	Aspirin therapy	6	71	<0.001
	ACEI/ARB therapy	7	57	<0.001
	Statin therapy	5	38	<0.001
	Aspirin + ACEI/ARB + Statin	2	20	<0.001
**QOF score**		45	61	<0.001

## Discussion

Our worldwide audit demonstrated a wide range in QOF scores across the 45 centres (range 10.16 to 90.14 out of a maximum score of 99), suggesting considerable variability in care quality, both in terms of care process performance and target process achievement. Standards were frequently sub-optimal. Centres in Europe achieved higher QOF scores than those based in the rest of the world. QOF scores demonstrated positive correlation with GDP and THE%, suggesting that diabetes care quality varies according to social-economic circumstances.

However some centres defied this trend. The centre ranked fifth overall (IND10) with 80.6 out of 99 points was joint thirty-second for GDP and classified as a low-to-middle income country by GNI. In contrast, the centre ranked forty-first overall with 19.7 out of 99 points (AUS1) was in a country ranked seventh highest for GDP and classified as a high income country by GNI. Therefore, provision of excellent diabetes care does not depend entirely on per capita income or total healthcare expenditure.

In the questionnaire study, the majority of colleagues and patients from the study centres were in support of the Alphabet Strategy checklist, agreeing that it was both practical and evidence based. Over 70% responded in the positive regarding adoption of the strategy in clinical practice, though just over half thought it could be applied in their economic background. Practically, it would obviously require translation to the local language. Indeed translations now exist in French, Telugu, Somali, and Gujarati. It was considered a useful aid to patient education with the patient information posters. Additionally, over two thirds of patient responders thought they should use the Alphabet Strategy themselves. However, there were a few reservations regarding its indirect costs - HbA1c, creatinine, and lipid profile assessments are expensive in countries with limited resources, and the costs of Guardian drug implementation especially with statins could be prohibitive to the patient.

Our pilot implementation study demonstrated significant improvements in care process performance with the use of the Alphabet Strategy over a period of four months [16]. With some modifications to adapt to local resources, the strategy checklist might be a tool to improve diabetes care in evolving economies such as India. We acknowledge however that there a wider barriers to effective diabetes treatments in developing countries such as political, cultural and social issues [17]. Health education is often under-resourced, and behaviours can be complex and difficult to change. Infrastructure is not set up to support chronic disease management. Specialist diabetes medical personnel are lacking as is the reliable provision of medicines and insulin.

We also acknowledge the difficulties associated with implementation of checklists in medicine. Following the implementation of the WHO Surgical Safety Checklist, several publications identified a range of barriers to the efficient performance and uptake of the process. These included confusion regarding its proper use, pragmatic challenges to efficient workflow especially in emergency surgery, regular access to resources in developing countries, and individual beliefs and attitudes [18]. For the latter, feedback reports found that nurses and anaesthetists were largely supportive of the process, but some surgeons were ‘not very enthusiastic’ [19]. Strategies identified for success included enlisting a local champion, good training and staff understanding, employing staff feedback to enable checklist modification, enhanced teamwork, and ownership by the local team.

Implementation of the Alphabet Strategy checklist at George Eliot Hospital was not without necessary background efforts to address organisational and cultural issues. At that time, there was a sole consultant diabetologist who designed the checklist and acted as local champion to the trust and local general practitioners. He was ably supported by an enthusiastic diabetes care team that sought to provide high quality care in a patient-centred care approach. Regular feedback and audit sessions gave continued impetus to the process as well as allowing modification of the checklist.

The benefits achieved by the Alphabet Strategy in our pilot study suggest that the checklist was successful in providing the technical solution to managing the complicated task of diabetes care. Implementing the checklist was probably aided by the fact that the care team was small and enthusiastic for the process, that modifications occurred to adapt to local resource availability, and that they were relatively unhindered by upper management input. Whether the Alphabet Strategy can be implemented in a larger organisation is uncertain. Potentially it provides a useful technical solution to the task of delivering comprehensive care as demonstrated by the overall consensus from the questionnaire study. Additionally, the Alphabet Strategy has been placed on the NICE website as an example of Shared Learning for the implementation of their Type 1 and Type 2 Diabetes guidelines [20].

Effective diabetes care is complicated, demanding on patients and healthcare professionals, and requires motivation and organisation. The philosophy of the Alphabet Strategy checklist is that health care should be ‘**POETIC**’:

- **P**ateint-centred, public health driven and professional inspired - patient-safe, meeting the needs of the community with emphasis on prevention
- **O**utcome-based - real health improvements base on clear and measurable outcomes
- **E**vidence-based - based on clinical research and informed by local clinical audit
- **T**eam-delivered - multidisciplinary, professionally inspired and fit for purpose
- **I**ntegrated - across all healthcare services, and related sectors and agencies
- **C**ost effective, cost efficient and clinically sound - high quality resource management and rigorous clinical audit.

Improved diabetes care will prevent or delay complications, improve quality of life and increase life expectancy. In the Steno-2 Study, there was clear evidence that high quality multifactorial intervention in diabetes care reduced all of these outcomes: all-cause mortality by 46%, cardiovascular death by 57%, end-stage renal disease by 83%, the need for retinal laser therapy by 55%, stroke by 85%, and amputations by 50% [21]. The Alphabet Strategy delivers effective clinical outcomes because health care professionals, users and carers all subscribe to a single systematic approach to diabetes management. All the elements of diabetes care are delivered in a coherent, consistent and timely fashion. Other Alphabet Strategy derived resources include patient care plans, education posters for each letter of the checklist, Ramadan advice (posters and care plans) and a full educational package of PowerPoint slides (available at http://www.abcdiabetes.co.uk). These materials are free to use in the public domain and hence has the potential for easy adaption.

Central to the POETIC vision of health care is the principle that all users should receive the best health service resources permit. No-one should be denied what is effective and affordable. The Clinical Guidelines Task Force of the International Diabetes Federation has identified appropriate standards of care for implementation in settings with widely different access to resources [22].

## Conclusions

Most people with diabetes reside in low and middle income countries. Wide variations in care quality were observed across the study centres with care often sub-optimal especially in non-high income countries. The Alphabet Strategy was considered favourably by the majority of persons participating in the questionnaire study with 71% of healthcare professions considering adopting the checklist in their daily practice. The checklist was implemented successfully in a low resource setting resulting in improvements in several aspects of care. The Alphabet Strategy can feasibly be adapted to different economic circumstances and has the potential to be implemented widely throughout the world.

## Electronic supplementary material

Additional file 1: **Alphabet Strategy questionnaire.** (PDF 71 KB)

Additional file 2: **Sample of answers to question 13.** (PDF 77 KB)

## Abbreviations

JRM: John Morrissey
VP: Vinod Patel
LV: Lakshminarayanan Varadhan
ADA: American diabetes association
BMI: Body mass index
EASD: European Association for the Study of Diabetes
GDP: Gross domestic product
GNI: Gross national income
IDF: International diabetes federation
LE: Life expectancy
NICE: National institute for health and care excellence
QOF: Quality and outcomes framework
THE%: Total health expenditure percentage per capita
VAS: Visual analogue scale
WHO: World health organization.
