# Supplementary material for: Quality of diabetes care worldwide and feasibility of implementation of the Alphabet Strategy: GAIA project (Global Alphabet Strategy Implementation Audit)
Source: BMC Health Serv Res. 2014 Oct 11;14:467. doi: 10.1186/1472-6963-14-467 (PMC4283094; doi:10.1186/1472-6963-14-467)
Supplement: Supplementary file 1 — Additional file 1: Alphabet Strategy questionnaire. (PDF 71 KB) [file 12913_2013_3553_MOESM1_ESM.pdf]

# Alphabet Strategy Questionnaire

Minimum of 5 per team including 2 patients and 3 healthcare professionals. Ideally fill 7

Please indicate your response along the bold line with a vertical mark.

1.

| **What do you think of the Alphabet Strategy approach?** | |
|---|---|
| Strongly Disagree | Strongly Agree |
| | |

2.

| **Are you likely to adopt this strategy in your clinical practice?** | |
|---|---|
| Strongly Disagree | Strongly Agree |
| | |

3.

| **Do you think your patients would understand and benefit from the educational potential of this strategy?** | |
|---|---|
| Strongly Disagree | Strongly Agree |
| | |

4.

| **Do you think this will improve the outcome of your practice?** | |
|---|---|
| Strongly Disagree | Strongly Agree |
| | |

5.

| **Do you think this strategy can apply to your economic background?** | |
|---|---|
| Strongly Disagree | Strongly Agree |
| | |

6.

| **Do you think this strategy needs to be translated in your local language?** | |
|---|---|
| Strongly Disagree | Strongly Agree |
| | |

7.

| **Do you think the English version will be more useful in your clinics?** | |
|---|---|
| Strongly Disagree | Strongly Agree |
| | |

8.

| **What do you think of the Diabetes Care Plan?** | |
|---|---|
| Not Useful | Very useful |
| | |

9.

| **What do you think of the educational posters?** | |
|---|---|
| Not Useful | Very useful |
| | |

10.

| **Do you think this strategy is practical?** | |
|---|---|
| Yes ☐ | No ☐ |

11.

| **Do you think this strategy is evidence based?** | |
|---|---|
| Yes ☐ | No ☐ |

12.

| **Which health care professional can adopt this strategy (√ for yes, X for no)** | | | | | |
|---|---|---|---|---|---|
| GP | Dietitian | Nurse | Surgeons | Administrators | Patients |
| ☐ | ☐ | ☐ | ☐ | ☐ | ☐ |

13.

**Please enter at least 3 comments you may have regarding the following questions**

13 A.

**General comments**

- 
- 
- 
- 
- 
- 
- 
- 

13 B.

| How could this strategy be implemented? |
| --- |
| •<br><br>•<br><br>•<br><br>•<br><br>•<br><br>•<br><br>•<br><br>• |
