# Supplementary material for: Quality of diabetes care worldwide and feasibility of implementation of the Alphabet Strategy: GAIA project (Global Alphabet Strategy Implementation Audit)
Source: BMC Health Serv Res. 2014 Oct 11;14:467. doi: 10.1186/1472-6963-14-467 (PMC4283094; doi:10.1186/1472-6963-14-467)
Supplement: Supplementary file 2 — Additional file 2: Sample of answers to question 13. (PDF 77 KB) [file 12913_2013_3553_MOESM2_ESM.pdf]

Alphabet Strategy questionnaire: Sample of answers to question 13

General comments-

This strategy seems to cover the whole spectrum. Nothing is missing. Pitched at a clear level.

Good strategy. The alphabet makes it easier for educators to remember all areas/ aspects to teach patients. The summary page is very useful. Some pictures may not be understood by patients but are useful.

A quick and useful way of remembering all to check for, especially in a busy Diabetes clinic.

Too much information for a patient to handle, especially statistics. Probably more useful for patients in a city rather than a town/village.

Posters are useful for both patients and their relatives/caregivers.

Applicable mostly in cities, it should be applicable to rural populations also.
Easy to read and understand. Easy to answer. Very practical questions.

Good care plan, which may be difficult to implement in my country (Grenada).

Very useful for diabetes patients; to solve problems for patients, to give more nursing care/doctor care to patients.

The diabetes care plan is definitely a useful way of monitoring patient profile.

Good system to ensure full check-up, as I feel health is the most important thing to me and my family. Money is no problem for me but it is for others in Punjab. I like the way there is more education.

Diabetes is very important, it affects a lot of my family. It is good to have a check-up but expensive.

Most patients are old and need motivation, especially type 2. Educational background and motivation should be taken into account when explaining. Educational posters are useful only if followed by discussions and questions session. Practical training of the patient is crucial.

Very good idea. Needs translating into Spanish. Very practical.

Good, simple strategy. Not affordable for patients here. Needs to be translated into Spanish and Quechua.

It is not possible to measure HbA1c and cholesterol in our lab (Tanzania). People have to pay for their medications thus purchasing guardian drugs and insulin will be a burden for them. Exercising is not really part of our culture.

Review BP Target. Would it be possible to have something specific for children or adolescents – type 1? You don't include urine dipstick tests for acetone etc
The posters for education are pictorial and are only missing the theory. The alphabet strategy helps us to remember the most important points.

The alphabet strategy facilitates memorisation. 'Guardian drugs' is a strong, robust term. Good system for global management. It may be used in primary or secondary prevention to communicate simple messages.

Good strategy. Increases the awareness of the public regarding diabetes care and complications. Easy checklist for diabetes care professionals. Proper motivation, adequate infrastructure and good economic background will definitely help this sort of strategy and care plan.

Very good. It will cover all aspects of the disease without missing any major areas of affliction. Simple to follow and easily reproducible.

How could this strategy be implemented?

Might need to be simplified. Would be useful to have a healthcare professional explaining the strategy to patients.

Need for translation into several languages in a multi-lingual society.

Information about different aspects by different health care professionals
Reinforcing different aspects about diabetes care at regular intervals. Poster, video-clippings useful for patients and family. Information booklet for the patient – basic aspect is a language one can understand.

Translate into tamil. Here no one can afford an HbAlc test/full lipid profile so it is not practical for us.

Not always practical as medication is not always available. Patients do not usually follow what they are told. The expense of medications makes it difficult to follow. Good plan but practically it might not work. Education is the most important route.
The educational part could be implemented by teaching the staff about the importance of good diabetes control, and by teaching this to the patients. Information booklets, posters etc could be displayed to make people more aware.

By involving all of the healthcare professionals. By making it practical and accessible. By promoting and educating. By knowing people with risk factors and acting rapidly.

Food choices will need to change to local culture. Will need to have more podiatrists for better foot care. This strategy can be implemented on a community level basis. Nurses and doctors who work at community clinics can use these posters to educate patients. The posters can be placed on display in out-patient clinics and other public buildings. Health professionals can also share this information at schools, churches etc. Media programmes can be used to educate the public.

Radio/TV information program. Make it policy at government level or hospital level.

Posters could be done in Hindi and English. Lab values on posters to be mg/dl. Foot care awareness needs more attention in our country (India) as patients ignore their feet totally. Diabetes in pregnancy has not been covered. In India we are regularly screening all pregnant women with a 758m glucose load (as we are ethnically more prone to develop diabetes). Currently this strategy is already in place except for a podiatrist. We would appreciate help setting up a podiatry clinic in our own hospital – protocol for foot examination and prevention of diabetic foot and foot care products.

Healthcare professionals should always revise patient education programme. Economically people cannot afford treatment. We want more health care professionals for this strategy.

Making posters in regional languages, make a package deal for patients that the cost of the package would be much more economical than the medical tests – also it would ensure that all tests are done.

Local language (Hindu and Punjab) and English care plan and posters. Make it convenient and value for money.

Punjabi or Hindu languages would be better – more information for me to read. Help with costs.

All different specialists should spend time advising patients and making sure they follow advice. More attention/education should be given to in-patients. Young clinicians have not realised how important educating the patient is. The first step is to give a general idea of the alphabet approach when the patient is hospitalised. Important not to overload the patients with information.

Advice and diet control should be absolutely personalised in terms of lifestyle, BMI, blood test.
Educational process is comprised of 7 sessions where their progress is monitored.
Leaflets are not enough. Education is more successful. 30% of compliance lower in socio-economic status.

Sometimes we spend less time for advising the patients. The clinical examination sometimes is not perfect from some doctors.

Funding required to enable patients to buy medications. Needs to be spread via journals or medical people.

Translate strategy into Spanish and also Quecha if possible. Lower drug prices to enable patients to afford strategy. Leaflets available for patients.

Not affordable for many patients. Needs to be easily accessible for our doctors (available in Spanish). Easy access for doctors and patients on the internet. Cheaper insurance or drug prices.

Coordinate it with present weekly diabetic clinic.

Translate into French – distribute to diabetology and cardiology services and to GPs.

Create associations for diabetics. Inform young patients as well as their guardians.
Inform people around diabetics. Distribute to diabetes services. Prevention in adolescents.

Posters in pharmacies, hospitals and surgeries.

Public awareness programs, setting up of diabetes care team, appropriate guidelines and regular medical education programmes for the general practitioners, facilities for follow up programmes.

Training of doctors to develop a national protocol incorporating the above strategy. Making administrators aware that treating a complication of diabetes costs much more than preventing the complication.

By following up the protocol in all diabetic patients, motivating and educating them regarding the disease and importance of adherence to therapy and other measures.
